# An evaluation of pharmacology curricula in Australian science and health-related degree programs

**DOI:** 10.1186/1472-6920-13-153

**Published:** 2013-11-19

**Authors:** Hilary Lloyd, Tina Hinton, Shane Bullock, Anna-Marie Babey, Elizabeth Davis, Lynette Fernandes, Joanne Hart, Ian Musgrave, James Ziogas

**Affiliations:** 1 The University of Sydney, Sydney NSW 2006, Australia; 2 School of Medical Sciences (Pharmacology), The University of Sydney, Sydney NSW 2006, Australia; 3 Gippsland Medical School, Monash University, Northways Road, Churchill VIC 3842, Australia; 4 School of Science and Technology, University of New England, Armidale NSW 2351, Australia; 5 Department of Pharmacology, Monash University, Clayton VIC 3800, Australia; 6 School of Medicine and Pharmacology, The University of Western Australia, Crawley WA 6009, Australia; 7 School of Medical Sciences, RMIT University, Bundoora VIC 3083, Australia; 8 Discipline of Pharmacology, School of Medical Sciences, University of Adelaide, Adelaide SA 5005, Australia; 9 Department of Pharmacology, University of Melbourne, Parkville VIC 3010, Australia

**Keywords:** Pharmacology, Curriculum, Education, Survey, Science, Medicine, Nursing, Pharmacy

## Abstract

**Background:**

Pharmacology is a biomedical discipline taught in basic science and professional degree programs. In order to provide information that would facilitate pharmacology curricula to be refined and developed, and approaches to teaching to be updated, a national survey was undertaken in Australia that investigated pharmacology course content, teaching and summative assessment methods.

**Methods:**

Twenty-two institutions participated in a purpose-built online questionnaire, which enabled an evaluation of 147 courses taught in 10 different degrees. To enable comparison, degrees were grouped into four major degree programs, namely science, pharmacy, medicine and nursing. The pharmacology content was then classified into 16 lecture themes, with 2-21 lecture topics identified per theme. The resultant data were analysed for similarities and differences in pharmacology curricula across the degree programs.

**Results:**

While all lecture themes were taught across degree programs, curriculum content differed with respect to the breadth and hours of coverage. Overall, lecture themes were taught most broadly in medicine and with greatest coverage in pharmacy. Reflecting a more traditional approach, lectures were a dominant teaching method (at least 90% of courses). Sixty-three percent of science courses provided practical classes but such sessions occurred much less frequently in other degree programs, while tutorials were much more common in pharmacy degree programs (70%). Notably, problem-based learning was common across medical programs. Considerable diversity was found in the types of summative assessment tasks employed. In science courses the most common form of in-semester assessment was practical reports, whereas in other programs pen-and-paper quizzes predominated. End-of-semester assessment contributed 50-80% to overall assessment across degree programs.

**Conclusion:**

The similarity in lecture themes taught across the four different degree programs shows that common knowledge- and competency-based learning outcomes can be defined for pharmacology. The authors contend that it is the differences in breadth and coverage of material for each lecture theme, and the differing teaching modes and assessment that characterise particular degree programs. Adoption of pharmacology knowledge-based learning outcomes that could be tailored to suit individual degree programs would better facilitate the sharing of expertise and teaching practice than the current model where pharmacology curricula are degree-specific.

## Background

The discipline of pharmacology embraces both the experimental and clinical sciences. Experimental pharmacology is vital to our understanding of drug action in the treatment of disease as well as to the pharmaceutical industry for drug discovery and development. Clinical pharmacology is essential for prescribing practice in medicine and nursing and underpins pharmacy practice and therapeutics. Pharmacology includes a number of branches such as pharmacodynamics, pharmacokinetics and therapeutics, and is taught in non-vocational, science degree programs and vocational, health professional programs. Currently, there are significant challenges and opportunities facing the teaching and learning of pharmacology. The applied nature of the discipline, the move towards integrated course structures, the deconstruction of discipline boundaries and increasing numbers of students seeking higher education raise issues concerning retention of subject-based discipline integrity, scope of material taught and maintenance of academic standards. These issues also provide opportunities to interact with other biomedical and clinical disciplines, educate a larger number and broader range of students, and to develop strategies to advance the discipline and adapt teaching and learning methods. Evaluation of current pedagogical approaches to pharmacology teaching in Australia is, therefore, not only an important first step towards addressing the aforementioned challenges, but will facilitate curriculum refresh and design. These are significant issues for stakeholders in the educational sector, the pharmaceutical industry and the health professions.

In the face of a growing movement to dismantle discipline boundaries, there is evidence from clinical practice that inadequate pharmacology knowledge is a growing concern, raising the question of the true role of disciplines and in particular the integrity of pharmacology as foundation material essential to safe and effective practice. In a survey conducted in Australia [1], only 13% of teaching staff agreed unequivocally that nursing graduates had sufficient knowledge for safe practice. In 2008, the Australian Medical Education Study (AMES) of medical students, junior doctors and educators/employees revealed that clinical pharmacology was one of the least successful components of medical education [2]. Indeed, only 39% of medical students considered that they were adequately or well prepared in clinical pharmacology. This finding reinforced that of a survey of graduating medical students in the UK in which deficiencies in the areas of basic pharmacology, pharmacokinetics and therapeutic drug monitoring were highlighted by 53-59% of the medical graduates surveyed [3]. Subsequently, Heaton and colleagues [4] published the views of 2413 UK medical students and recent graduates on prescribing preparation; the majority view was that teaching and assessment in this area was inadequate. A study undertaken by Dornan and co-workers [5] demonstrated the gravity of the situation when it was determined that there was an error rate of 8-10% in prescription writing. These studies indicate significant deficiencies in medical pharmacology education even though learning outcomes for pharmacology in medical curricula are apparently well represented [6].

Comparable concerns have been raised by the biopharmaceutical industry. The Pharmaceuticals Education Council (PEC) of Australia, which was set up in 2003 to investigate pharmaceutical workforce needs, reported that employers were concerned about a lack of job-ready graduates skilled in drug discovery and development. To address this issue, the PEC recommended that pharmacology and other biomedical sciences be introduced into postgraduate courses [7].

The deficiencies highlighted above in teaching and perceptions of learning of pharmacology raise an initial vital question: “*what and how*” are we teaching pharmacology? Surprisingly few studies have addressed this. In 1990, Walley and colleagues [8] conducted a survey of pharmacology teaching in medical degrees in the UK based on 17 areas of core information, 16 core skills and 5 core attitudes that were regarded as representing a core curriculum for clinical pharmacology [9]. They found that there was good agreement about the importance of certain areas (e.g. prescribing for the elderly, management of overdose and adverse drug reactions) and that these areas were widely taught (85-100% of all degrees surveyed); nevertheless, there was considerable variation in how extensively other areas were taught (e.g. efficacy and toxicity of non-prescription drugs, taught by 35%).

In 1996, the Education Sub-committee of the British Pharmacology Society (BPS) conducted a survey of the content of Bachelor of Science (BSc) pharmacology courses [10]. Significant variation in pharmacology course content was found across the universities surveyed, with only a limited number of topics being taught in depth. In a second survey conducted in that same year, 78% of teaching was found to be chalk and talk lectures, reflecting a traditional approach to pharmacology teaching [11]. Arising out of these findings was a call by the BPS for a pharmacology core curriculum for BSc degree programs to parallel the development of a core curriculum in clinical pharmacology for medical programs. One clear advantage of a core curriculum is that it can address the issue of content overload, where content is added *ad hoc* but nothing is removed; a phenomenon referred to as ‘coveritis’ [12,13].

In recent years, the BPS and the Australasian Society of Clinical and Experimental Pharmacologists and Toxicologists (ASCEPT) have created pharmacology core curricula for science, medicine, dentistry, nursing and veterinary science courses, with each curriculum developed for a single program. In the USA, the Association for Medical School Pharmacology published *The Knowledge Objectives in Medical School Pharmacology*[14] and the International Union of Basic and Clinical Pharmacology (IUPHAR) has, through its website, made available 35 pharmacology curricula used in pharmacy, medicine, and science degree programs in different institutions and countries. In 2008, the Association of American Medical Colleges (AAMC) released a report on safe and effective prescribing practices, which includes six tables containing the broad learning objectives ideally taught and evaluated at medical schools [15]. In a recent version of Tomorrow’s Doctors [16], published by the General Medical Council (UK), there is guidance on knowledge pertaining to basic and clinical pharmacology for doctors.

Surveys previously undertaken have compared the “*what and how*” of pharmacology teaching in specific degree programs at different institutions [1,10,17,18]. To our knowledge, no study has compared the “*how*” as well as the “*what*” of pharmacology teaching across different degree programs at a national level. The purpose of our study was to investigate content and teaching methodology across degree programs in order to compare curricula. Specifically, this study was designed to provide a snapshot of the current approaches to the delivery and assessment of pharmacology content, rather than student learning, across courses and programs in which pharmacology was taught (at Bachelor and Masters levels) at higher education institutions across Australia. Our view is that commonalities in content and approach have the potential to better facilitate the sharing of expertise and teaching practice, benchmarking and curriculum renewal activities.

## Methods

### Participants

Of the 39 higher education institutions in Australia, 37 were identified that teach pharmacology and contact was made with relevant personnel in each institution after ethics approval had been obtained. Of the 37 institutions contacted, 27 agreed to participate (73% response rate) and of those, staff from 22 completed the survey (81% completion rate) (Table 1). Participating institutions formed a representative sample of universities in Australia, including research-intensive (Group of Eight), Australian Technology Network and regional universities, and all degree programs into which pharmacology is taught. Since the breadth of degree programs are also taught across the institutions that did not participate, our data are generalisable to those institutions. Individual participants were full- or part-time academic staff members who were fluent in English (as a first or second language). Informed consent was implicit in completion of the survey.

**Table 1:** Participating institutions and academic staffing levels

**Institution**	**Number of full time academic staff teaching pharmacology**
Australian National University	4
Bond University	5
Charles Darwin University	1
Deakin University	1
Flinders University	4
Griffith University	3
James Cook University	2
La Trobe University	2
Monash University	12
Murdoch University	3
Queensland University of Technology	5
RMIT University	9
University of Adelaide	7
University of Melbourne	11
University of Notre Dame	3
University of South Australia	6
University of Southern Queensland	2
University of Sydney	13
University of Technology, Sydney	2
University of Tasmania	3
University of Western Australia	9
University of Western Sydney	1

### Terminology

Definitions of the following terms are provided in Table 2 for clarification: degree program, course, theme, topic, stand-alone course, and integrated course.

**Table 2:** Definitions of key terms*

**Term**	**Definition**
**Degree program**	One of four core areas of study, namely science, pharmacy, nursing and medicine for which a university degree is obtained
**Course**	Credit point unit of study within a degree. Some institutions refer to this as a subject or unit
**Theme**	A broad, primary concept within pharmacology and toxicology. For the purpose of this article, themes are drawn from lecture content
**Topic**	A specific, secondary concept within a theme. For the purpose of this article, these topics are drawn from lecture content
**Stand-alone course**	A course dedicated entirely to the discipline of pharmacology as an independent field of study
**Integrated course**	A course in which pharmacology is one of many disciplines taught using a blended approach to the content

*Terminology occasionally varied substantially between institutions that contributed to this study. Key terms were chosen based on frequency of use amongst institutions and the potential for generalisability of terminology across institutions in other countries.

### Survey instrument

The online questionnaire consisted of 18 open- and closed-ended questions, with multiple response categories. The questionnaire was designed by the project team to provide insight into the pedagogical approaches to the delivery of pharmacology content during the years 2008-9 offered within science, pharmacy, nursing and medicine Bachelor and Masters degrees in Australia. The questionnaire was divided into five parts: A - E. The time required to complete the questionnaire varied from one to several hours depending on the number of pharmacology courses offered by a given institution and the number of staff members available to assist with survey completion.

To begin, demographic data about pharmacology teaching at participating institutions was solicited. Part A of the questionnaire sought information about the total number of full-time academic staff employed, the number of academic staff teaching pharmacology, the year level(s) when pharmacology was taught, the numbers of students enrolled in courses containing pharmacology and whether those courses were delivered in a stand alone or integrated mode.

The breadth and coverage of pharmacology content were addressed in Part B of the survey. Sixteen lecture themes were identified within the course content, and each theme was sub-divided into a number of lecture topics. The categorisation of fifteen key themes and topics was developed using a combination of pharmacology principles, systems-based pharmacology and disease-based therapeutics referenced against the Tables of Contents of a selection of commonly used pharmacology texts and clinical reference handbooks; for example, pharmacokinetics is a theme, for which drug metabolism is a topic within that theme. The appropriateness of the list of themes and topics was verified and finalised in a pilot study of pharmacology courses from eight institutions. Only two lecture topics were identified for some lecture themes, while up to 21 were identified for others. The list of lecture themes and topics was then incorporated into the questionnaire (see Table 3). Survey participants were asked to reference their responses against this list in order to standardise the collected data. The sixteenth theme was classified *Miscellaneous* to capture topics that failed to fit within the previous 15 themes. The topics identified within this theme were derived *post hoc* from survey responses and are included in Table 3.

**Table 3:** Lecture topics provided for each lecture theme in the survey

**Lecture theme**	**Lecture topic**
Pharmacodynamics	Introduction to Pharmacology/Drug Action
	Drug-receptor interactions
	Drug targets (receptors, enzymes, transporters)
	Quantitation of drug action/Measurement in Pharmacology
	Agonism/Agonists
	Antagonism/Antagonists
	Models of drug action/Pharmacodynamics
	Determinants of drug potency/effectiveness
	Physicochemical properties of drugs
	Receptors (ion channels, GPCRs, intracellular receptors)
	Intracellular signalling/Signal transduction
	Transporters
	Desensitisation/Tachyphylaxis
Pharmacokinetics	Drug absorption and distribution
	Drug metabolism
	Drug excretion
	Pharmacokinetics applied
	Pharmacokinetics calculations
	Effects of age/renal disease/liver disease on pharmacokinetics
	Pharmacogenetics/Pharmacogenomics
Xenobiotic metabolism and toxicology	Xenobiotic metabolism
	Target organ toxicology
	Teratology/Teratogenesis/Reproductive toxicology
	Genotoxicity/Cancer-causing agents
	Poisons/Chemical warfare/Pesticides
	Venoms/Biological warfare
	Poisonous plants
	Environmental toxicology/Environmental pollutants
	Adverse drug reactions/Idiosyncratic drug reactions
	Drug interactions
	Pharmacoepidemiology/Epidemiology
Drug design and development	Drug discovery/design and development
	Molecular modelling/Computer-aided drug design
	Structure-activity relations
	Research methods in Pharmacology (pre-clinical)
	Intellectual property commercialisation in Pharmaceuticals
	Clinical trials
	Pharmacoeconomics
	How drugs reach the market/Drug registration
	Therapeutic drug monitoring/Regulatory affairs/Drug laws
Neuropharmacology	Autonomic nervous system
	Cholinergics (parasympathetic/neuromuscular junction)
	Adrenergics (sympathetic)
	Autacoids (Histamine, 5-HT, Eicosanoids)
	Central nervous system
	Neuropeptides, proteins and purines (cannabinoids, purines, endogenous opioids, NPY, substance P, etc.)
	Neurotransmitters (catecholamines, indoleamines, amino acids, ACh, etc.)
	Neuroendocrinology
	Schizophrenia/Antipsychotics
	Mood/Depression/Antidepressants/Mood stabilisers
	Anxiolytics/Sedatives/Hypnotics
	Epilepsy/Seizures/Antiepileptics/Anticonvulsants
	Dementia/Drugs for dementia/Drugs for cognition
	Motor control/Movement disorders/Drugs for movement disorders
	Neurodevelopment/Neurodegeneration
	Multiple sclerosis (and demyelinating diseases)/Drugs for multiple sclerosis (and demyelinating diseases)
	Neuroprotective agents
	Excitotoxicity
	Arousal and stimulants/Drugs for ADHD
	Behavioural neuropharmacology/Psychopharmacology
	Mydriatics/Miotics
Respiratory drugs	Drugs for asthma (bronchodilators, corticosteroids, LT antagonists, cromolyns etc.)
	Antitussives
	Drugs for COPD
Cardiovascular and renal drugs	Drugs for hypertension
	Drugs for heart failure
	Drugs for dyslipidaemia/hypercholesterolaemia
	Drugs for angina/Myocardial infarction
	Drugs for cardiac arrhythmias
	Anticoagulants/Antiplatelet drugs/Thrombolytics/Fibrinolytics
	Diuretics
Gastrointestinal drugs	Drugs for dyspepsia, reflux and peptic ulcers
	Drugs affecting gastrointestinal motility
	Antinauseants/Antiemetics
	Drugs for inflammatory bowel disease
Musculoskeletal drugs	Drugs for gout and hyperuricaemia
	Drugs affecting bone and calcium metabolism
	Treatment of osteoporosis
Metabolic/endocrine/genital drugs	Antidiabetics/oral hypoglycaemics
	Drugs for fertility/Contraception
	Anti-oestrogens/Anti-progestogens
	Drugs for labour/Uterine drugs
	Corticosteroids/Drugs for adrenal disorders
	Androgens and anabolic steroids
	Drug treatment of impotence and prostate disease
	Hormone replacement therapy/Oestrogens
	Thyroid and antithyroid drugs
	Drugs for obesity, energy balance and appetite
	Drugs for pituitary disorders
Chemotherapy	Antibacterials
	Antivirals
	Anthelminthics/Antiprotozoals
	Antifungals
	Cancer/Neoplasia
	Cancer chemotherapy
Analgesia/Anaesthesia/Anti-inflammatories	Nociception/Pain mechanisms
	Local anaesthetics
	General anaesthetics
	Opioid analgesics
	Migraine/Drugs for migraine
	Inflammation
	Steroidal anti-inflammatories
	Nonsteroidal anti-inflammatories and paracetamol
	Immunosuppressants/DMARDs
	Drugs for hay fever/anaphylaxis/antihistamines
	Neuromuscular blockers
Complementary medicines	Plant-sourced drugs
	Complementary medicines
	Herbal medicines
	Foods and beverages as drugs
Future therapies	Gene therapy/RNA therapies
	Antibody therapies
Drugs of abuse	Addiction/Drug dependence
	Drugs of abuse (Cocaine, LSD, MDMA, Cannabis, Heroin, Alcohol, BZs etc.)
	Pharmacological management of addiction
	Drugs in sport
	Nootropic agents
Miscellaneous	Drugs for liver disease
	Ethics
	Drugs for eczema, psoriasis and acne
	Drugs for anaemia
	Information sources about drugs
	Equity of access to drugs
	Drugs and the oral cavity
	Prescription writing and the PBS
	Other (as specified by participants)
	- statistics
	- communication skills
	- free radicals and cell damage
	- social aspects of drug abuse
	- medication management
	- medication safety
	- polypharmacy
	- legal issues
	- drug advertising

Information about the different types of teaching methods used (e.g. lectures, wet and computer laboratories and tutorials) and the time spent utilising each method was obtained in Part C. The summative assessment methods employed and the relative proportion of in-semester versus end-of semester assessment was obtained in Part D. Information about formative assessment methods was not collected in the survey. Finally, Part E addressed the type, level and frequency of course evaluation.

Initially, a trial questionnaire was created using a formatted Excel spreadsheet. Instructions guided respondents on how to complete the questionnaire: to choose from a drop down menu (with pre-coded response options) or to fill in the space provided. Amendments to navigation and ease of use were made in the pilot study, in addition to changes required with respect to the verification of themes and topics. The finalised questionnaire was scripted in Microsoft Word (National Field Services, Melbourne, Australia) and further iterations and amendments were made during the scripting process. The final questionnaire was hosted online using SPSS software (SPSS Inc., Chicago, Illinois, USA) by National Field Services (Australia). In accordance with the requirements of the project funding body, the Australian Learning and Teaching Council, the questionnaire is openly available on request.

### Data collection and ethics approval

Ethics approval was obtained from Human Research Ethics Committees of participating institutions of the project team. The ethics approval numbers were: 11-2008/11375 for the University of Sydney and MUHREC CF09/0656: 2009000215 for Monash University. Human Research Ethics Committees at the University of Western Australia, the University of Melbourne, the University of Adelaide, RMIT and James Cook University granted approval for this study based on ethics approval obtained from University of Sydney in accordance with their respective policies allowing for the recognition of ethics approval by an external Human Research Ethics Committee and/or in tandem with the ethics approval for a companion survey of student perceptions on teaching. Following this, letters were sent to Heads of Department/School of all higher education institutions identified in which pharmacology was taught inviting participation in the survey and requesting contact details of a nominated liaison person for that Department/School. Liaison individuals were then contacted by email or phone and provided with details about the survey, participant information and questionnaire instructions. Follow-up contact was made via email two and three weeks later to all liaison persons to remind them of the survey completion date. The liaison person completed and/or requested that academic staff within their School/Department complete the questionnaire.

### Data analysis

Questionnaire data were exported from SPSS data files to Microsoft Excel spreadsheets and analysed. The original study examined a number of degree programs in addition to those described here into which pharmacology is taught, such as dentistry, optometry and veterinary medicine, however only the major four (science, pharmacy, nursing and medicine) are presented here. Descriptive statistics from this pooled data were extracted and reported, including frequency counts, percentages, means, medians and ranges.

### Breadth and coverage of pharmacology content in different degree programs

A key aspect of the data analysis was to determine the breadth of lecture themes and lecture topics taught in the different degree programs, and to obtain an index of the coverage of each. The breadth of content was defined in two ways: 1) the identification of lecture themes taught in each course and the percentage of courses in a given degree program that taught each of those themes; and 2) the percentage of courses that addressed a particular topic within a theme.

The extent of coverage of each topic is defined as the average number of lectures devoted to the topic within a theme. This is represented as an index of content coverage using the following calculation:

$$\frac{\text{average number of topic lectures per theme}}{\left(\text{total number of topics per theme}\right)\;\times \;\left(\text{fraction of topics taught per theme}\right).}$$

A resultant index greater than 1 represents greater than 1 lecture per topic within a given theme.

## Results

### Context in which pharmacology is taught

There was great diversity in the characteristics of the 22 institutions that participated in the survey in terms of staff numbers (1-13), staff:student ratios, total student enrolment (4,000–40,000), and location (urban and regional). Across these 22 institutions, 147 courses were identified into which pharmacology was taught, representing 10 different degrees (7 non-vocational, 3 vocational). For the purposes of analysis, the 10 degrees were grouped into four distinct degree programs (Table 4).

**Table 4:** Specific degrees included for each degree program

Degree program*	**Degrees included**	Number of courses**	**Number of institutions**
**Science**	Science, Medical Science, Biomedical Science, Pharmaceutical Science, Pharmaceutical Engineering, Health Science, Biotechnology	59	14
**Pharmacy**	Pharmacy	41	10
**Nursing**	Nursing	24	11
**Medicine**	Medicine	27	10

*While many Australian institutions diversify their degree offerings, for example, offering a Bachelor of Science as well as a Bachelor of Biomedical Science and a Bachelor of Biotechnology, this is not true of all institutions, nor is this necessarily generalisable to institutions outside Australia. Choice of categories was based in part on the recognition that programs for which a student might obtain a discrete named degree in Australia might constitute a major or focus of a more generally-named degree elsewhere (e.g. a major in Biotechnology is found in some Bachelor of Science degrees). It should be noted that the Medicine degree category does not distinguish between graduate-entry and undergraduate programs.

**Note that 4 courses were accounted for twice, for example, taught to Science and Pharmacy.

A further 10 courses were identified into which pharmacology is taught across 13 degree programs. Given the diversity of structure, scope and duration of these varied degree programs, to merge them into a single category that might be called allied health would potentially mask important differences in approaches to delivery, breadth of content and assessment. Therefore, this dataset was not included in the analysis presented here.

**Figure 1:**
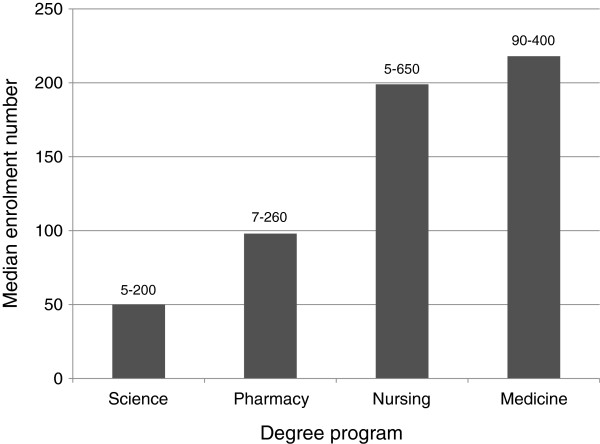
**Student enrolment numbers for courses within each degree program.** Data are represented as median enrolment number; ranges are given above each bar.

The median student enrolment numbers for courses that taught pharmacology for each degree program varied widely (Figure 1), with medicine and nursing having the largest cohort sizes and science having far fewer students enrolled. Given the marked cohort size range within certain degree programs, the median provides a better representation of a potentially skewed distribution. The smaller cohort sizes (5-20) mainly represent the inclusion of specialised courses (e.g. advanced practice and research-based courses) within each degree program. Across programs, pharmacology was taught most often at second and third year levels in science and pharmacy programs, whereas in nursing and medicine it was taught mainly at first and second year level (Table 5).

**Table 5:** A comparison of the timing of pharmacology delivery across degree programs

**Year of degree**	**Science (n** = **59)**	**Pharmacy (n** = **41)**	**Nursing (n** = **24)**	**Medicine (n** = **27)**
**1st year**	3.5%	2.4%	25%	25.9%
**2nd year**	27%	21.9%	54.2%	29.6%
**3rd year**	61%	41.5%	0	18.5%
**4th year**	6.5%	24.4%	4.2%	11.1%
**5th year**	0	9.7%	16.7%	7.4%
**6th year**	0	0	0	3.7%
**Not specified**	2%	0	0	0

Data are expressed as the number of courses offered in a given year as a percentage of total courses offered in each program, where n = number of courses surveyed per degree program (as identified in Table 4).

Note: In Australia, basic science and nursing programs are of 3 years duration; Pharmacy programs are 4 years; and Medicine programs can be 4, 5 or 6 years, with undergraduate programs of longer duration. Later years are likely to be at Honours level or equivalent.

**Figure 2:**
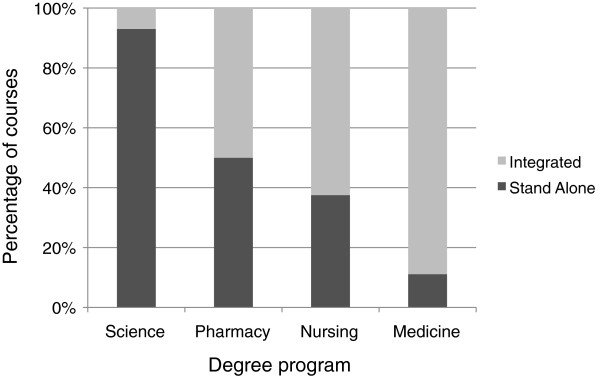
**A comparison of the nature of the delivery of pharmacology across degree programs.** Each course within a degree program was evaluated to determine whether pharmacology was presented as stand-alone content or was integrated within that course with material that crossed a number of disciplines, of which pharmacology was only one.

The difference in approaches to the delivery of pharmacology is reflected in a comparison of the extent of teaching integration across degree programs (Figure 2). In science degree programs, pharmacology was taught mainly as one or more stand-alone course(s), whereas in medicine and nursing the pharmacology content was more likely to be integrated across a number of courses or with other discipline material within courses. By contrast, the teaching of pharmacology was evenly split between stand-alone and integrated courses in pharmacy at the institutions surveyed.

### Breadth and coverage of pharmacology content in different degree programs

All but one of the 16 lecture themes (Table 3) were taught in each of the degree programs. The exception is Future Therapies, which was not covered in any of the nursing courses surveyed (Table 6). The lecture themes analgesia/anaesthesia/anti-inflammatories, pharmacokinetics and pharmacodynamics were amongst the most widely distributed and were taught in 50% or more of the courses surveyed in all degree programs, with the exception of pharmacy. As can be seen in Table 6, there was notable variation across degree programs for certain lecture themes.

**Table 6:** A comparison of course content by lecture theme across degree programs, depicted as frequency of lecture themes taught (percentage) across degree programs

Lecture theme in rank* **order**	**Mean (%)**	**Science (n = 59) (%)**	**Pharmacy (n = 41) (%)**	**Nursing (n = 24) (%)**	**Medicine (n = 27) (%)**
**Analgesia/Anaesthesia/Anti-inflammatories**	53 ± 10	51	42	67	52
**Pharmacokinetics**	51± 10	58	37	58	52
**Pharmacodynamics**	50 ± 16	63	27	58	52
**Neuropharmacology**	45 ± 2	47	42	46	44
**Cardiovascular and Renal Drugs**	42 ± 10	37	32	54	44
**Chemotherapy**	39 ± 17	31	20	58	48
**Metabolic/Endocrine/Genital Drugs**	39 ± 10	41	32	33	41
**Xenobiotic Metabolism and Toxicology**	37 ± 5	36	27	50	41
**Drugs of Abuse**	34 ± 9	34	22	42	37
**Respiratory Drugs**	32 ± 6	31	24	38	33
**Drug Design and Development**	32 ± 17	56	24	21	26
**Miscellaneous**	28 ± 9	24	24	21	41
**Gastrointestinal Drugs**	22 ± 9	15	15	33	26
**Complementary Medicines**	22 ± 2	20	22	25	22
**Musculoskeletal Drugs**	19 ± 7	14	24	13	26
**Future Therapies**	13 ± 13	10	10	0	30

Error in the "Mean" column represents the standard deviation. Courses were evaluated for the presence or absence of material taught covering each theme. An individual course might cover some, all or none of the identified themes and therefore, a stand-alone pharmacology course might be expected to be represented in each percentage whereas an integrated course might be represented only in a single percentage within a degree program.

*Ranking is based on the mean frequency of lecture themes taught (percentage) across degree programs from highest to lowest.

*n* = number of courses surveyed.

The percentage of lecture topics taught within each associated lecture theme was determined for the four degree programs (Table 7). As indicated by this parameter, lecture topics that addressed a given theme were more broadly covered in medicine degree programs (median percentage 48%). On average fewer topics per theme were addressed in science and nursing programs. No one degree program covered all topics across all themes, though the number of missing topics varied between programs with 5 topics not addressed in lectures for science and 6 for medicine as compared to 19 topics not addressed in nursing.

**Table 7:** A comparison of the number of lecture topics per theme across degree programs

**Lecture theme (total no. topics per theme)**	**Science (n = 59) (%)**	**Pharmacy (n = 41) (%)**	**Nursing (n = 24) (%)**	**Medicine (n = 27) (%)**
Pharmacokinetics (7 topics)	37 ± 4	40 ± 7	29 ± 6	52 ± 9
Analgesia/Anaesthesia/Anti-inflammatories (11 topics)	30 ± 4	41 ± 6	32 ± 6	45 ± 9
Pharmacodynamics (13 topics)	36 ± 4	45 ± 11	26 ± 7	36 ± 9
Neuropharmacology (21 topics)	27 ± 3	31 ± 4	31 ± 5	28 ± 7
Cardiovascular and Renal Drugs (7 topics)	51 ± 6	75 ± 11	54 ± 10	76 ± 10
Chemotherapy (6 topics)	46 ± 6	54 ± 12	45 ± 10	61 ± 10
Xenobiotic Metabolism and Toxicology (11 topics)	31 ± 4	25 ± 6	22 ± 5	34 ± 8
Metabolic/Endocrine/Genital Drugs (11 topics)	23 ± 5	58 ± 9	23 ± 5	35 ± 9
Drugs of Abuse (5 topics)	44 ± 4	40 ± 8	42 ± 7	36 ± 7
Respiratory Drugs (3 topics)	44 ± 5	53 ± 13	48 ± 9	52 ± 8
Drug Design and Development (9 topics)	31 ± 4	24 ± 4	13 ± 5	37 ± 8
Miscellaneous (9 topics including “other”)	10 ± 2	12 ± 2	29 ± 10	26 ± 6
Gastrointestinal Drugs (4 topics)	39 ± 10	75 ± 14	59 ± 11	57 ± 12
Complementary Medicines (4 topics)	44 ± 9	22 ± 4	29 ± 6	67 ± 16
Musculoskeletal drugs (3 topics)	25 ± 8	57 ± 10	22 ± 11	67 ± 16
Future Therapies (2 topics)	58 ± 12	63 ± 18	0	50 ± 6
**MEDIAN TOPIC PERCENTAGE**	**37**	**43**	**29**	**48**

Data are expressed as average frequency ± s.e.m. For the courses identified in Table 6 that addressed pharmacology themes, each course was evaluated to identify the topics covered within each theme. A percentage of coverage of each topic was then determined for those courses that addressed the theme. The higher the number, the greater the number of topics covered in a theme. As an example, 58% of courses addressed pharmacokinetics as a theme in Science degree programs (Table 6), the proportion of those courses that addressed the topic of drug metabolism was determined, followed by drug excretion and so forth, then averaged for topics in the theme.

The median percentage coverage across the topics within each theme is also provided for overview.

**Figure 3:**
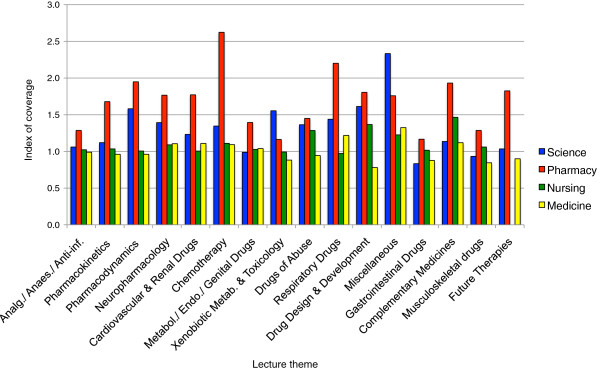
**A comparison of index of coverage across degree programs.** The index of coverage indicates the number of lectures allocated to a lecture theme relative to the number of topics taught. Data are expressed as: $\frac{\mathrm{average}\;\mathrm{number}\;\mathrm{of}\;\mathrm{topic}\;\mathrm{lectures}\;\mathrm{per}\;\mathrm{theme}}{\left(\mathrm{total}\;\mathrm{number}\;\mathrm{of}\;\mathrm{topics}\;\mathrm{per}\;\mathrm{theme}\right)\;\times \;\left(\mathrm{fraction}\;\mathrm{of}\;\mathrm{topics}\;\mathrm{taught}\;\mathrm{per}\;\mathrm{theme}\right)}$. Lecture themes ranked from left to right on the basis of mean lecture theme percentage across degree programs from highest to lowest. An index of greater than 1 represents more than 1 lecture taught per lecture topic.

The number of lectures on topics within each theme was calculated to provide an index of coverage in each degree program (Figure 3). This analysis revealed that in pharmacy most lecture themes had a greater number of lectures per theme, compared to other degree programs. In science degree programs some themes (e.g. Drugs of Abuse, Drug Design & Development) had similar coverage to that in pharmacy. Overall, there were fewer lectures per theme in medicine and nursing when compared to science and pharmacy programs.

### Course delivery

The various methods of course delivery and the time allocated to each method for each of the degree programs were compared (Table 8). These included both traditional (lectures, practicals and tutorials) and non-traditional (computer simulations, workshops, problem-based learning (PBL) tutorials, online) teaching approaches, as well as project work. Non-traditional teaching methods were less frequently used compared with traditional approaches.

**Table 8:** A comparison of the type of, and time allocated to, teaching methods across degree programs

**Teaching method**	**Science (n = 59)**	**Pharmacy (n = 41)**	**Nursing (n = 24)**	**Medicine (n = 27)**
Lectures^1^	90%	90.2%	91.7%	100%
*hours allocated*	26 (4 to 48)	36 (0 – 60)	12 (3 – 50)	12 (5 – >60)^4^
Practicals (wet labs)^2^	62.7%	19.5%	4.2%	3.7%
*hours allocated*	8 (1 to 48)	7.5 (3 – 15)	4	3
Computer modelling	11.8%	4.9%	0	0
*hours allocated*	7 (4 to 18)	2.5 (2 – 3)	0	0
Computer simulations	22%	14.6%	0	11%
*hours allocated*	6 (3 to 27)	4 (3 – 6)	0	2 (1 – 3)
Tutorial	44%	70.7%	50%	40.7%
*hours allocated*	5 (1 to 33)	12 (2 – 54)	10 (3 – 16)	4 (2 – >60)
Computer tutorial	13.5%	7.3%	0	3.7%
*hours allocated*	3.5 (1 to 8)	2 (2 – 3)	0	1
PBL tutorial	11.8%	0%	8.3%	37%
*hours allocated*	4 (1 to 16)	0	4.5 (3 – 6)	8 (3 – 12)
Workshop	11.8%	31.7%	8.3%	14.8%
*hours allocated*	6 (1 to 21)	12 (2 – 24)	9	4 (3 – 36)
Online	8.5%	9.7%	12.5%	3.7%
*hours allocated*	19 (2 to all)^3^	22 (2 – 36)	>60 (30 – >60)^4^	2
Project	39%	26.8%	4.2%	3.7%
*hours allocated*	12 (2 to >60)^4^	10 (2 – 10)	18	10

Frequency of courses that utilise the listed teaching methods is expressed as a percentage of total number (n) of courses surveyed in each program. Hours allocated are expressed as median number of hours with ranges in brackets.

*Note*:

^*1*^*Science courses that do not utilise lectures*: *one is online and four are Honours courses. Of the four Pharmacy courses that do not utilise lectures*, *two are fourth year courses and two are professional practice courses. The two Nursing courses that do not utilise lectures are fully online courses*.

^*2*^*The term* “*practicals*” *is synonymous with laboratory classes*.

^*3*^‘*all*’ *indicates that the entire course is delivered online*.

^*4*^>*60 refers to greater than 60 hours spent using the particular teaching method*, *and is generally indicated for Honours* (*research only*) *courses as well as online courses*.

The overwhelming majority (90% or above) of courses continue to use lectures, although the number of hours allocated to lectures varied considerably. Of time devoted to activities other than lectures, practical classes still dominated in science degree programs, with 63% of courses offering wet-labs (ranging from 1-48 hours). A third of courses in science also offered computer-based practicals (i.e. computer modelling (12%) and computer simulation (22%)). Strikingly, it was rare for courses in non-science degree programs to utilise practical sessions for pharmacology teaching. Tutorials predominated in pharmacy (70%) relative to other degree programs but none were identified as PBL tutorials. Medicine programs showed a much higher percentage of courses that used PBL tutorials (37%). In the nursing degree program two fully online courses were included in the survey; this accounts for greater than 60 hours allocated to this method of course delivery. Pharmacology research projects were offered in one-quarter to one-third of all pharmacy and science courses but in only 4% of courses in nursing and medicine.

### Summative assessment in pharmacology

Various types of in-semester summative assessment were used across the degree programs (see Table 9) with science and pharmacy programs using all formats identified to some extent. In science, the most common format was laboratory reports (24% of courses using this form of in-semester assessment), whereas pharmacy courses were more likely to use assignments (20%), reflecting the difference in the use of practicals (wet labs) in each degree program. By contrast, nursing and medicine were more likely to employ pen-and-paper quizzes in preference to other methods (30% and 31% respectively).

**Table 9:** A comparison of in-semester summative assessment formats across degree programs

**Assessment type**	**Science (n = 59)**	**Pharmacy (n = 41)**	**Nursing (n = 24)**	**Medicine (n = 27)**
Laboratory report	23.9%	7.4%	2.5%	0
Research Project	10.3%	4.6%	0	6.2%
Essay	4.5%	8.4%	2.5%	0
Quiz: pen-and-paper	9.7%	13.7%	30%	31.2%
Quiz: online	1.9%	1%	7.5%	0
Assignment	14.2%	20%	12.5%	15.6%
Exercise	7.1%	11.5%	10%	0
Presentation (oral or poster)	13.5%	16.8%	5%	3.1%
Thesis	1.3%	1%	0	0
Thesis defence	1.3%	1%	0	0
Other	12.3%	14.7%	30%	43.7%

Data are expressed as frequency (percentage) of courses using in-semester summative assessments surveyed in each program.

*n* = number of courses surveyed.

For medicine and nursing the summative assessment format that comprised the highest percentage belonged to the category called *Other* suggesting that the list provided for participants was not sufficiently comprehensive. For pharmacy this category represented the second most common format, while for science it was the fourth most common. For science, activities in this category included assessment of data, critiquing of papers, journal club presentation and participation, participation in group discussions, mid-semester examinations, literature reviews, and enquiry-based learning case reports. For pharmacy, this included workbook learning activities, clinical cases, and drug reports. Other assessment types in nursing included those listed for pharmacy, as well as mid-semester examinations. Finally, for medicine *Other* assessment types included workplace supervisor reports, National Prescribing Service case studies, online open book assessment of tutorials, and problem-based learning tutor marks.

By contrast to the high degree of variability of in-semester assessment, the style of end-of-semester examination was much more conserved across degree programs (Table 10). The primary format for theory examinations combined multiple-choice questions (MCQ) with short answer questions (SAQ) or essays, with between 51-81% of courses employing this format. Additionally, practical examinations in the form of objective structured clinical examinations (OSCEs) were utilised for those degree programs requiring application of clinical practical skills.

**Table 10:** A comparison of end-of-semester examination styles across degree programs

**End-of-semester exam type**	**Science (n = 59)**	**Pharmacy (n = 41)**	**Nursing (n = 24)**	**Medicine (n = 27)**
Theory examination: only MCQ	5.3%	4.8%	9.5%	15.1%
Theory examination: only SAQ or Essay	38.6%	11.9%	4.8%	3%
Theory examination: mix of MCQ/SAQ or Essay	50.9%	66.6%	81%	60.6%
Practical examination: mix of MCQ/SAQ or Essay	1.8%	2.4%	0	3%
Practical examination: OSCE	0	14.3%	0	12.1%
Other	3.5%	0	4.8%	6.1%

Data are expressed as percentage of total end-of-semester examinations surveyed for each degree program.

*n* = Number of courses surveyed.

*MCQ* = Multiple-choice question.

*SAQ* = Short-answer question.

*OSCE* = Objective structured clinical examination.

**Figure 4:**
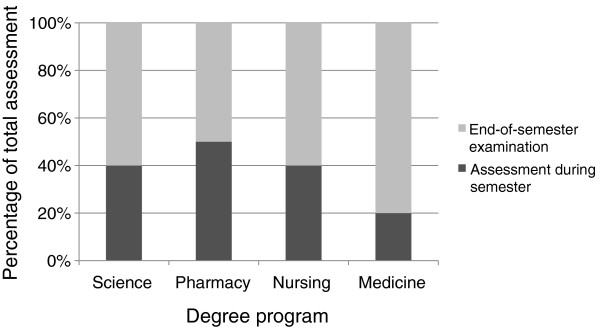
**A comparison of the relative proportion of in-semester and end-of-semester assessment across degree programs.** Data are expressed as the median percentage of assessment across the courses surveyed in each degree program.

These similarities in assessment are reinforced when the proportion of assessment during semester was compared with the value of the end-of-semester examination (Figure 4). In general, the end-of-semester examination was worth 50-80% of the overall grade regardless of the program evaluated. Eight percent of courses surveyed had no end-of-semester examination (4 science degree programs, 5 pharmacy degree programs, 3 nursing degree programs) and 6% of courses were assessed by end-of-semester examination alone (9 medicine degree programs).

### Communication in pharmacology courses

Web-based facilities for communication between staff and students were widely utilised across courses and degree programs (96% science, 100% pharmacy, 100% nursing, 100%, medicine). The main roles for this communication were online announcements, email group lists and online discussions.

### Evaluation of pharmacology teaching

In all degree programs with the exception of medicine, the most common method employed to obtain student feedback on teaching consisted of a mix of structured and open-ended questions. In medicine, evaluation consisted primarily of a structured question format. Evaluations were generally carried out at the university level and conducted once per semester across all degree programs.

## Discussion

In the current study, we undertook a national survey of the current practice of pharmacology teaching and summative assessment. We determined the breadth and coverage of pharmacology curricula across four different degree programs, allowing content, teaching approaches and summative assessment activities to be compared.

### Defining the current approach to the pharmacology curricula

Data was obtained from 147 courses, taught in 10 different degrees, which were grouped into four different degree programs (Table 4); data from these courses were then collated to provide a snapshot of pharmacology teaching across the degree programs. The focus of this survey was to provide an overview of the current approaches to the delivery of content, types and weighting of in-semester versus end-of-semester summative assessment and breadth and coverage of topics within the field of pharmacology. While examination of the weighting of each assessment task within each degree program as well as various types of formative assessment would have been useful, this facet was not directly addressed in this project.

A difference was noted in the timing of the delivery of pharmacology content between degree programs. More than half of the medicine courses and three-quarters of nursing courses concentrated pharmacology teaching to the first and second years of their respective programs, recognising that medical degrees in Australia differ in duration depending on the course entry level offered (i.e. undergraduate degrees into which students can enter either directly from secondary school and is 5-6 years duration or after completing at least one university degree for which the medical degree is of 4 years duration) and nursing degrees have a 4 year duration. Pharmacy and science degree programs, by contrast, have courses towards the middle to latter stages (second and third years) of their respective programs (pharmacy 63%; science 88%), with the majority of pharmacy degrees of 4 years duration, and science programs 3 years (4 years for a science honours degree). The differences in timing for medicine and nursing compared with other degree programs may be associated with one or more of the following factors: the early timing of clinical placements in nursing; the division of the medical degree into pre-clinical and clinical years; that Australian medical degrees are delivered as either undergraduate or postgraduate degrees with different degree durations; and that pharmacology is being increasingly taught as part of an integrated curriculum. By contrast, pharmacology is often offered for the first time at the second year level in science following completion of pre-requisite first year sciences such as chemistry, mathematics and biology.

In order to evaluate pharmacology course content, 16 lecture themes were developed. The sixteenth theme (*Miscellaneous*) covered a mixture of topics while the other 15 lecture themes correlated closely with the subject areas provided in the survey of pharmacology courses in BSc degrees (UK) [10] and US medical schools [14]. While clinical pharmacology was not identified as a theme in our survey, perusal of Table 3 shows that aspects of clinical pharmacology were represented across the lecture themes. Respondents also identified prescription writing as a specialised topic within the *Miscellaneous* theme.

Although there was a high level of congruence with respect to lecture themes across degree programs (Table 6), such that content boundaries were broadly similar, there were notable differences in coverage (as represented by the calculated index) and to a lesser extent the breadth of lecture themes (Table 7 and Figure 3). For example, more time was devoted to drug design and development in science degree programs than that provided to students enrolled in medicine degree programs. Overall, the index of coverage indicated that pharmacy students were provided with more lecture hours across the majority of pharmacology topics than students in other degree programs (Figure 3). However, the breadth of topics covered in nursing degree programs (84% of topics) was less than that in pharmacy, medicine or science (92% of topics for pharmacy; 95% for medicine; 96% for science (data not shown)). The survey design did not allow the index of theme coverage to be considered equivalent to the depth in which the theme was taught, and it is recognised that a greater number of lectures allocated to topics within a theme does not necessarily indicate that the theme was taught in greater depth.

Lectures were the most common mode of teaching in all degree programs thereby justifying the use of lecture themes and lecture topics to identify curriculum content. However, caution must be exercised when using lecture themes to reflect course content, as the methods of delivery differed widely across degree programs. For example, in science degree programs, practical classes were common whereas PBL tutorials (or a variation of this mode) were relatively frequent in medicine programs and rare in science degrees. Thus, the observation that teaching coverage is generally lower for medicine must be viewed in the context of the integrated approach in medical schools in which PBL tutorials or similar types of teaching approaches address topics and themes in a non-traditional manner. Furthermore, teaching within an integrated course may allow for more focused teaching whereas in stand-alone courses time may be spent providing context and relevant physiological/pathophysiological background. Nevertheless, it is reasonable to propose that there is close alignment between content taught in lectures with content taught using other modes of teaching so that lecture themes and topics would be expected to be a fair approximation of course content.

The predominance of lectures in so many programs is noteworthy, given the recent trend to move away from such a traditional method of delivery. As mentioned, the use of PBL and other non-traditional teaching methods is more common in the health-related fields when compared with the science degree programs. The reasons for which lecture delivery have been retained is/are unclear, although it should be acknowledged that the format of the lectures was not explored in this survey; thus it is possible that they varied from didactic to more interactive discussion-based styles. Additionally, the efficiency with which curriculum content may be delivered to large numbers of students should not be discounted. The demonstration that lectures form a major proportion of teaching may offer a valuable opportunity for curriculum reconsideration, renewal and redesign.

### Pedagogic approach, graduate destination and discipline integrity

While lectures were the most common mode of delivery of course content, clear differences emerged with respect to additional modes of teaching and types of assessment across degree programs. For example, in science degree programs scientific enquiry, laboratory skills and research methodology were emphasised, as seen by the inclusion of laboratory work, laboratory reports and thesis writing. On the other hand, tutorials and workshops predominated in the vocational, health-related degree programs where clinical reasoning skills were accentuated and OSCEs used. Thus, how pharmacology is taught and the types of assessment used to evaluate the learning, reflect graduate destinations.

Pharmacology is taught both in stand-alone courses and in integrated curricula across degree programs and institutions. In science degree programs pharmacology was more likely to be taught in stand-alone courses, whereas in medicine approximately 90% of pharmacology was taught within integrated curricula, with other vocational health-related degree programs following a similar trend to that of medicine. Arguably, discipline integrity and expertise is most readily retained and developed within stand-alone courses, thus science degree programs may have a hitherto unrecognised and important role to play in this regard. On the other hand, in health-related degrees interdisciplinary teaching in an integrated curriculum may be advantageous to facilitate the acquisition and integration of knowledge from a range of biomedical sciences as well as a team-based approach to patient management. Within integrated curricula, however, vigilance is required to ensure pharmacology is well represented in order to foster, for example, safe prescribing and lifelong learning [19].

### Recognition of foundation principles and content boundaries of pharmacology

Concerns raised about the level of pharmacology knowledge on the part of medical and nursing graduates [1-5], as well as from industry [7], argue for maintenance of discipline integrity to facilitate curriculum renewal and redesign to address these deficiencies. While defining core knowledge in pharmacology has received considerable attention [8-10,14,15], levels of knowledge and competencies may be better defined by criteria expressed as learning outcomes [20]. Evaluation of the data from our survey suggests that broad, knowledge-based learning outcomes could be developed for key principles of pharmacology that outline a minimum or threshold standard of knowledge and competency for each degree program. These would address questions of pharmacology content boundaries [21] and reflect the central role that pharmacology principles play in drug discovery, development and registration processes and in the safe and effective use of medicines by health professionals. It is envisaged that these learning outcomes would be constructed with input from stakeholders in higher education, pharmaceutical industry and health professions. Further, we suggest that development of knowledge-based learning outcomes for graduates of pharmacology would be of value to course designers and regulators involved in the evaluation of various other allied health degree programs seeking an expansion of their pharmacological roles, such as to obtain limited or extended prescription rights.

## Conclusion

The survey provides an overview of current approaches to pharmacology teaching and summative assessment in science, pharmacy, nursing and medicine degree programs in Australia and identifies a high degree of congruence of curriculum content between these degree programs. We believe that the observed similarities and differences in pharmacology teaching, content and methods across degree programs can inform curriculum renewal and development. This provides the basis and rationale for development of knowledge-based learning outcomes for pharmacology that can inform pharmacology teaching across degree programs with minimum standards articulated for each degree program. Since the identity of pharmacology is not readily defined [22-24], defining what needs to be taught in differing degrees assumes an importance hitherto not fully appreciated. We suggest that the development of broad, knowledge- and competency-based learning outcomes for pharmacology would lead to greater collaboration between experimental and clinical pharmacologists in the teaching of this important discipline to students in science, pharmacy, nursing and medicine degree programs.

## Abbreviations

AAMC: Association of American Medical Colleges; AMES: Australian Medical Education Survey; ASCEPT: Australian Society of Clinical and Experimental Pharmacologists and Toxicologists; ASPET: American Society for Pharmacology and Experimental Therapeutics; BPS: British Pharmacology Society; BSc: Bachelor of science; IUPHAR: International union of basic and clinical pharmacology; MCQ: Multiple-choice questions; OSCE: Objective structured clinical examinations; PBL: Problem-based learning; PEC: Pharmaceuticals Education Council of Australia; SAQ: Short-answer questions.

## Competing interests

The authors declare that they have no competing interests.

## Authors’ contributions

JZ, HL, SB, AMB, ED, IM and LF conceived of the study. All authors participated in the design and coordination of the study. TH, HL and SB designed the survey tool. TH, HL, JZ, ED, IM, LF and AMB piloted the survey. TH coordinated dissemination of survey and acquisition of data and analysed all data. TH, HL and SB drafted the manuscript. TH, HL, SB, JZ, ED, IM, LF, AMB and JH critically revised the manuscript. All authors have given final approval of the version to be published.

## Authors’ information

Hilary Lloyd and Tina Hinton are designated dual first authors.

## Pre-publication history

The pre-publication history for this paper can be accessed here:

http://www.biomedcentral.com/1472-6920/13/153/prepub

## References

[B1] Bullock S Manias E The educational preparation of undergraduate nursing students in pharmacology: a survey of lecturers’ perceptions and experiences J Adv Nurs 2002 40 7 16 10.1046/j.1365-2648.2002.02335.x 12230523

[B2] Lawson M Bearman M Medical education in Australia: what makes for success? Summary report: surveys, focus groups and interviews 2007 Australia: Final report on PRN9183 & PRN9255 for the Department of Education, Science and Training

[B3] Tobaiqy M McLay J Ross S Foundation year 1 doctors and clinical pharmacology and therapeutics teaching. A retrospective view in light of experience Br J Clin Pharmacol 2007 64 363 372 10.1111/j.1365-2125.2007.02925.x 17506779 PMC2000658

[B4] Heaton A Webb DJ Maxwell SRJ Undergraduate preparation for prescribing: the views of 2413 UK medical students and recent graduates Br J Clin Pharmacol 2008 66 128 134 10.1111/j.1365-2125.2008.03197.x 18492128 PMC2485268

[B5] Dornan T Ashcroft D Heathfield H Lewis P Miles J Tully M Wass V An in depth investigation into causes of prescribing errors by foundation trainees in relation to their medical education – Equip study 2009 Manchester: Final report

[B6] Bull S Mattick K What biomedical science should be included in undergraduate medical courses and how is this decided? Med Teach 2010 32 360 367 10.3109/01421590903434144 20423252

[B7] Pharmaceuticals Education Council Report on Skills Gaps in the Pharmaceutical and Biopharmaceutical Industries. Phases 1- 3 2007 http://www.pharmacouncil.com.au/resources.php: accessed 19th August, 2012

[B8] Walley T Bligh J Orme M Breckenridge A Clinical pharmacology and therapeutics in undergraduate medical education in the UK: the future Br J Clin Pharm 1994 37 137 143 10.1111/j.1365-2125.1994.tb04253.x PMC1364590 8186060

[B9] Nierenberg DW A core curriculum for medical students in clinical pharmacology and therapeutics. The Council for Medical Education in Clinical Pharmacology and Therapeutics Clin Pharmacol Ther 1990 48 606 610 10.1038/clpt.1990.201 2249370

[B10] Dewhurst DG Page CP A survey of the content of BSc courses in pharmacology in UK universities - is it time for a core curriculum? Trends Pharmacol Sci 1998 19 262 265 10.1016/S0165-6147(98)01187-0 9703758

[B11] Markham T Jones SJ Hughes I Sutcliffe M Survey methods of teaching and learning in undergraduate pharmacology within UK higher education Trends Pharmacol Sci 1998 19 257 262 10.1016/S0165-6147(98)01221-8 9703757

[B12] Guilbert J-J Coveritis: an acute and chronic faculty disease Educacao Medica 1995 6 2 7

[B13] Achike FI Ogle CW Information overload in the teaching of pharmacology J Clin Pharmacol 2000 40 177 183 10.1177/00912700022008838 10664924

[B14] Eisenberg R, Rosenfeld G Knowledge Objectives in Medical Pharmacology Association for Medical School Pharmacology Chairs 2008 5 Updated version: http://www.amspc.org/pharmacology-resources/

[B15] Association of American Medical Colleges Contemporary issues in medicine education in safe and effective prescribing practices 2008 Washington DC: Medical School Objectives Project

[B16] General Medical Council Tomorrow’s doctors – outcomes and standards for undergraduate medical education 2009 United Kingdom http://www.gmc-uk.org/TomorrowsDoctors_2009.pdf_39260971.pdf

[B17] Jaillon P Teaching basic and clinical pharmacology to medical students: a 2006 survey in French Schools of medicine Therapie 2006 61 439 446 10.2515/therapie:2006071 17243273

[B18] O’Shaughnessy L Haq I Maxwell S Llewelyn M Teaching of clinical pharmacology and therapeutics in UK medical schools: current status in 2009 Br J Clin Pharm 2010 70 143 148 10.1111/j.1365-2125.2010.03665.x PMC2909818 20642558

[B19] Achike FI The temporal and challenging faces of integration in medical education: the fate of pharmacology Indian J Pharmacol 2011 43 227 231 10.4103/0253-7613.81492 21713082 PMC3113370

[B20] Australian Qualifications Framework First Edition July 2011 http://www.aqf.edu.au/wp-content/uploads/2013/05/AQF-1st-Edition-July-2011.pdf

[B21] Stupans I Evidence-based learning design: The case of pharmacology teaching in pharmacy programs 2010 University of New England: University Learning and Teaching Futures Colloquium http://www.une.edu.au/__data/assets/pdf_file/0007/52549/ULT-Futures-2010-Stupans.pdf

[B22] Black JW TiPS on identity Trends Pharmacol Sci 1996 17 121 10.1016/0165-6147(96)81585-9 8984737

[B23] Csaky TZ Is there an identity crisis in medical school pharmacology? J Med Educ 1976 51 935 937 978705

[B24] Vallance P Smart TG The future of pharmacology Brit J Pharmacol 2006 147 S304 S307 16402118 10.1038/sj.bjp.0706454 PMC1760753

